# Phylogeny, habitat together with biological and ecological factors can influence germination of 36 subalpine *Rhododendron* species from the eastern Tibetan Plateau

**DOI:** 10.1002/ece3.3874

**Published:** 2018-03-01

**Authors:** Yongji Wang, Liming Lai, Hui Du, Lianhe Jiang, Fei Wang, Chao Zhang, Ping Zhuang, Yuanrun Zheng

**Affiliations:** ^1^ Shanxi Normal University Linfen Shanxi China; ^2^ Key Laboratory of Resource Plants West China Subalpine Botanical Garden Institute of Botany Chinese Academy of Sciences Xiangshan Beijing China

**Keywords:** altitude, habitat, phylogeny, plant height, *Rhododendron*, seed germination, seed mass, Tibet

## Abstract

The reproductive stages of the life cycle are crucial in explaining the distribution patterns of plant species because of their extreme vulnerability to environmental conditions. Despite reported evidence that seed germination is related to habitat macroclimatic characteristics, such as mean annual temperature, the effect of this trait in controlling plant species distribution has not yet been systematically and quantitatively evaluated. To learn whether seed germination can predict species distribution along altitude gradients, we examined germination data of 36 *Rhododendron* species in southeastern Tibet originating from contrasting altitudes, habitats, plant heights, seed masses, and phylogenies. Germination varied significantly with altitude, habitat, plant height, and phylogeny and was higher in the light than in the dark. Germination percentage was highest at 10:20°C in the light and 15:25°C in the dark. As altitude increased, germination percentages first rose and then decreased, being highest at 3,500–4,000 m. Germination percentage and rate were highest on rocky slopes, increasing as seed mass and plant height rose. Variations in germination percentage and rate were not significant at subgenera, section, and subsection levels, but they were significant at species level. The results suggested that the relationship between germination and altitude may provide insights into species distribution patterns. Further, germination patterns are a result of long‐term evolution as well as taxonomic constraints.

## 1 INTRODUCTION

Seed germination represents an important life stage and can influence community dynamics (Zeng, Wang, Baskin, & Baskin, 2014; Zhou et al., 2015), lifetime fitness (Dukic, Dunisijevic‐Bojovic, & Samuilov, 2014), and life history characteristics (Wang et al., 2012). It is essential to study seed germination when examining reproductive strategies and evolutionary ecology (Baskin & Baskin, 2014a). Seed germination, seed mass, and mode of dispersal of wild plant species are heritable and contribute to seedling survival and are thus subjected to natural selection (Chen, Xu, Wang, & Mao, 2013; Zhou et al., 2015). A large proportion of studies focused on seed mass variation with altitude, latitude, habitat, and other environmental factors. Seed mass had a significant relationship with latitude across 2,706 species in global scale, with seed mass being significantly lower toward two holes. Seed mass declines significantly with latitude within‐species, but more slowly than cross‐species decline (Moles & Westoby, 2003). Seed mass varies significantly at different ecological levels, including among species, within‐species, and even within individuals. The reason for the results was that low temperature at high elevation may reduce photosynthetic rates, and short growing season may the time for seed development and seed provisioning (Baker, 1972; Moles & Westoby, 2004). Some studies found a negative correlation between seed mass and altitude (Baker, 1972; Bu et al., 2007). By contrast, a positive correlation was found between seed mass and altitude between congeneric lowland and alpine species, among species and within‐species. The explanation was that natural selection should favor the production of larger seeds in species at higher altitude (Guo, Mazer, & Du, 2010; Mazer, 1990). Even there had been so many studies on seed mass, little studies had focused on the seed germination variation. Therefore, research on the ecological dynamics of seed germination can provide information about natural selection's role in germination dynamics. Differences in seed germination among species are connected to environmental factors, for example, rainfall, temperature, altitude, and light (Qu, Baskin, Wang, & Huang, 2008; Walck, Baskin, & Baskin, 1997; Zheng et al., 2005), and to life history traits as well (Li, Li, Dong, & Liu, 2011; Wang, Baskin, Cui, & Du, 2009; Wang et al., 2012) and phylogeny (Wang et al., 2009). Thus, phylogeny, biological, and ecological factors must be considered when examining natural selection's effect on the germination of seeds (Baskin & Baskin, 2014a). Many studies had proved that species that have near relative had conservative germination strategies. According to recent evidence, within a family or a genus, reproductive characters, such as seed morphology and germination, could be phylogenetic constrains. This shows seed germination can be stable evolutionary trait which constrains interspecific variation in germination characters (Rosbakh & Poschlod, 2015; Stromberg, Butler, Hazelton, & Boudell, 2011; Wang et al., 2009, 2014). Community‐level research is not common, but there is some information for tropical forest (Armstrong & Westoby, 1993; Bu et al., 2007; Dainese & Sitzia, 2013; Hodkinson et al., 1998; Murray, Brown, Dickman, & Crowther, 2004; Thompson & Hodkinson, 1998), temperate rain forest (Miller & Miller, 2011), alpine meadow (Bu et al., 2007), and arid environments (Stromberg et al., 2011). There is no information to date on subalpine forests, which differ physically and biologically from the other types of environments studied.

Nest model analysis of variances (ANOVAs) and phylogenetic comparative techniques have been used to examine the connection between environmental conditions and life history traits. Phylogenetically independent contrasts (PICs) are the most frequently used for such studies (Garland & Ives, 1992), as they can remove the outcomes of shared evolutionary histories by computing contrasts in trait values between sister taxa. If a difference in a trait significantly covaries with a contrast in another trait, then the traits are evolutionarily correlated (Armstrong & Westoby, 1993), but the independent contrast method can be difficult and is thus underused (Armstrong & Westoby, 1993).

The east Himalaya was regarded as the main center of evolution and distribution for *Rhododendron* species (Fang et al., 2005; Wang et al., 2014). The complicated environments provide various habitats for *Rhododendron* species, which might have various adaptive strategies. Therefore, *Rhododendron* species in this region can be used to understand the relationship between species traits, evolution, and environment.

The aim of this research was to study how life history, phylogeny, and ecological conditions affect seed germination of 36 subalpine *Rhododendron* species in the east Himalayas using ANOVAs and GLM. The comparative analysis allows one to identify the primary aspects influencing seed germination of subalpine forest species, thus adding to research on reproductive strategies and evolutionary ecology (Wang et al., 2012). We wanted to determine whether there were differences in germination strategies of *Rhododendron* species from subalpine areas compared to those from other habitats. We also wanted to determine the extent that life history and phylogeny qualities, such as seed mass, affect seed germination across species.

## 2 MATERIALS AND METHODS

### 2.1 Study sites and sampling methods

*Rhododendron* (Ericaceae) contains nine subgenera and more than 1,000 species and is one of the largest genera of angiosperms being broadly spread throughout Asia, Europe, and North America (Fang et al., 2005). Species are found in habitats ranging from latitudes 65°N to 20°S in tropical, temperate, and boreal zones. Species are found at altitudes from a few hundred meters to ~5,500 m above sea level, and this includes subtropical mountain evergreen broad‐leaved forests, coniferous and mixed broad‐leaved forests, coniferous forests, open coniferous forests elfin forests, and *Rhododendron* shrubs. *Rhododendron* plants and seeds morphologically vary significantly with altitude.

The Himalayas are the highest mountain range globally and consist of many ecological environments. From low to high elevation, the vegetation shifts from tropical to subtropical to temperate to alpine. There exist 351 species in three subgenera, six sections, and 41 subsections, a total of 36% of the species in the genus, in the Himalayan region in China. This region is also the center of diversity for the genus and is taxonomically complex (Dainese & Sitzia, 2013). *Rhododendron* species grow from a few hundred meters to ~5,500 m in this region, thus providing ideal conditions for studying variation.

The study locations are found on the southeastern Tibetan Plateau (27.239º–29.996ºN, 88.5–97.287ºE) near the Himalaya and Hengduan mountains, in altitudes ranging from 2,280 to 4,540 m and including Milin, Motuo, Bomi, Cuona, Longzi, Yadong, Linzhi, and Chayu counties. In 2010, seeds of 36 *Rhododendron* species from 48 populations in three subgenera, three sections, and 23 subsections were collected from habitats including alpine shrub, rocky slope, and forest. Seeds were obtained by hand in late September to early October from more than ten individual plants, selected randomly from three to five subpopulations at each altitude. We then pooled the seeds of each species and subpopulation, and we determined the mean seed mass of each species within a population at each altitude. Three to five mature, yet, unopened fruits were taken from each plant. To decrease any variation among individuals due to the possible effects of fruit position on seed mass, we took fruits at the basal, middle, and distal positions on each sampled inflorescence. The seeds were dissected from the fruit and air‐dried in groups of 1,000 until used. Three groups per population site were weighed to the nearest 0.0001 g on an electronic balance. The height of each sampled plant was measured to the nearest dm.

### 2.2 Germination experiments

**Figure 1:**
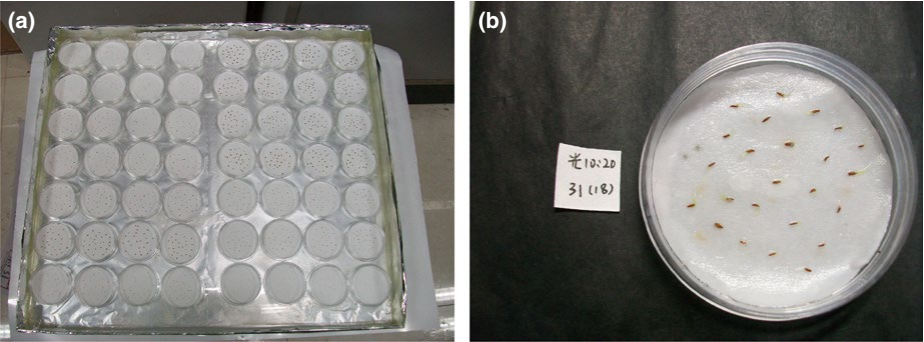
(a) Seeds of fourteen kinds of *R.s* before experiment. (b) Seeds of *R. hookeri* in germination

Germination experiments were performed in automatic temperature‐, light‐, and humidity‐controlled growth chambers equipped with cool white fluorescent light with a 12‐hr photoperiod (Figure 1a). Seeds were surface sterilized with 0.52% sodium hypochlorite solution for 1 min then rinsed several times with distilled water to prevent fungal infection (Zeng & Zheng, 2014; Zheng et al., 2014). Seeds were germinated (in 5 replicate treatments of 30 seeds each) in 90 × 15 mm Petri dishes with three layers of filter paper. Distilled water was added until the filter papers were saturated, and seeds were just afloat but not inundated. Emergence of the radicle was measured as germination and seeds were removed upon germination (Figure 1b). A trial was considered finished when the germination percentage stopped increasing in 60 days.

The effects of temperature and light on germination were determined using five alternating temperature regimes: 5/15, 10/20, 15/25, 20/30, and 25/35°C (night 10 hr: day 14 hr) in a randomized complete block design. The five temperature regimes closely approximated spring and summer germination conditions on the Tibetan Plateau in China. Two light/dark regimes were applied, comprising a 14‐hr photoperiod (photosynthetic photon flux density = 125 μmol m^−2^ s^−1^), nearly dark, in which germination was checked daily under dim light (photosynthetic photon flux density = 9.8 μmol m^−2^ s^−1^).

### 2.3 Attribute groups

Phylogenetic group: To evaluate the phylogenetic effect on seed germination, each of the 36 angiosperm species was assigned to an order according to the Angiosperm Phylogeny Group (APG) III system of plant classification (Angiosperm Phylogeny Group, 2009), a molecular‐based system of plant taxonomy (Table 1). A nested ANOVA model was used to examine the effect of taxonomic on FPG variation, and component *R* ^2^ values were calculated by the proportion of type sums of squares (SS) explained by each taxonomic level. This described how much were PFG variation corrected with each level nested within the superior level. Then, one‐way ANOVA models were fitted to the data set to examine divergence within versus among taxonomic groups.

**Table 1:** Environment variables and seed mass (Mean ± *SD*,* n* = 3, 1,000 seeds for each replicate) of 44 populations of 36 congeneric *Rhododendron* species

Population	Species	Altitude	Habitat	Mean height of plant (m)	Subgenus	Section	Subsection	Seed mass (g)
1	*R. vellereum*	4,220	1	2	1	1	10	0.0713 ± 0.0006
2	*R. tsariense*	4,170	1	1.3	1	1	21	0.0550 ± 0.0002
3	*R. catacosmum*	4,130	3	3	1	1	2	0.1161 ± 0.0005
4	*R. calvescens*	3,600	3	4	1	1	6	0.1491 ± 0.0031
5	*R. principis*	3,760	3	3.5	1	1	10	0.0772 ± 0.0017
6	*R. trichocladum*	3,660	3	1	3			0.0757 ± 0.0006
7	*R. hookeri*	3,680	2	1	1	1	12	0.0829 ± 0.0017
8	*R. megalanthum*	3,210	2	2.5	1	1	12	0.0536 ± 0.0019
9	*R. megalanthum*	2,600	2	2	1	1	12	0.0560 ± 0.0066
10	*R. maddenii* subsp*. Crassum*	2,650	2	2	2	2	13	0.1257 ± 0.0031
11	*R. maddenii* subsp. *Crassum*	2,670	3	2.5	2	2	13	0.1499 ± 0.0008
12	*R. arboreum* var. *roseum*	2,510	3	4	1	1	16	0.0207 ± 0.0047
13	*R. setiferum*	3,570	3	3	1	1	6	0.0940 ± 0.0005
14	*R. coriaceum*	3,260	3	3	1	1	11	0.0543 ± 0.0021
15	*R. kongboense*	4,450	1	0.1	2	3		0.0817 ± 0.0009
16	*R. keysii*	2,900	3	4	1	1	8	0.0703 ± 0.0007
17	*R. grande*	2,760	2	3	1	1	1	0.1764 ± 0.0106
18	*R. heliolepis*	3,420	3	1.5	2	2	15	0.0522 ± 0.0017
19	*R. polylepis*	3,570	3	1	2	2	20	0.0858 ± 0.0027
20	*R. lulangense*	3,170	2	1.5	1	1	10	0.1222 ± 0.0006
21	*R. bainbridgeanum*	4,000	1	1	1	1	6	0.0764 ± 0.0006
22	*R. trichostomum*	4,490	1	0.3	2	3		0.0828 ± 0.0014
23	*R. agastum*	3,570	3	1.7	1	1	7	0.0685 ± 0.0011
24	*R. erosum*	3,140	3	4	1	1	5	0.0993 ± 0.0062
25	*R. triflorum*	3,150	3	2	2	2	20	0.2845 ± 0.0048
26	*R. arboreum*	3,140	3	6	1	1	16	0.0718 ± 0.0001
27	*R. pruniflorum*	4,170	1	1.1	1	1	19	0.0263 ± 0.0015
28	*R. sinogrande*	2,640	2	5	1	1	1	0.1980 ± 0.0224
29	*R. pendulum*	2,870	3	1.2	2	2	14	0.0607 ± 0.0007
30	*R. mekongense*	3,940	1	0.4	3			0.0418 ± 0.0011
31	*R. mekongense*	3,690	1	1.5	3			0.0793 ± 0.0013
32	*R. campylogynum*	4,350	1	0.1	2	2	22	0.0193 ± 0.0009
33	*R. calostrotum var. calciphilum*	4,150	1	0.2	2	2	18	0.0235 ± 0.0006
34	*R. kyawi*	3,210	2	3	1	1	4	0.1069 ± 0.0017
35	*R. kyawi*	2,920	3	3.5	1	1	4	0.0978 ± 0.0055
36	*R. aperantum*	4,010	1	0.3	1	1	2	0.0393 ± 0.0008
37	*R. nivale*	4,450	1	0.1	2	2	9	0.1046 ± 0.0005
38	*R. aganniphum*	4,530	1	1.4	1	1	10	0.0689 ± 0.0003
39	*R. aganniphum*	4,170	1	1	1	1	10	0.1201 ± 0.0500
40	*R. hirtipes*	4,130	3	2	1	1	6	0.1002 ± 0.0008
41	*R. campanulatum*	4,040	1	2	1	1	3	0.0972 ± 0.0016
42	*R. campanulatum*	3,570	3	2	1	1	3	0.1060 ± 0.0006
43	*R. uvarifolium*	3,150	3	2	1	1	17	0.0967 ± 0.0058
44	*R. uvarifolium*	3,600	3	3	1	1	17	0.1182 ± 0.0009

Habitat: 1, alpine shrub; 2, rocky slope; 3, forest.

Subgenus: 1, Subgen. *Hymenanthes* (Blume) K. Koch; 2, Subgen. *Rhododendron*; 3, Subgen. *Pseudazalea* Sleumer.

Section: 1, Sect. *Ponticum* G. Don; 2, Sect. *Rhododendron*; 3, Sect. *Pogonanthum* G. Don.

Subsection: 1, subsect. *Grandia* Sleumer; 2, subsect. *Neriiflora* Sleumer; 3, subsect. *Campanulata* Sleumer; 4, subsect. *Parishia* Sleumer; 5, subsect. *Barbata* Sleumer; 6, subsect. *Selensia* Sleumer; 7, subsect. *Irrorata* Sleumer; 8, subsect. *Cinnabarina* (Hutch.) Sleumer; 9, subsect. *Lapponica* (Balf. F.) Sleumer; 10, subsect. *Taliensia* Sleumer; 11, subsect. *Falconera* Sleumer; 12, subsect. *Thomsonii* Sleumer; 13, subsect. *Maddenia* (Hutch.) Sleumer; 14, subsect. *Edgeworthia* (Hutch.) Sleumer; 15, subsect. *Heliolepida* (Hutch.) Sleumer; 16, subsect. *Arborea* Sleumer; 17, subsect. *Fulva* Sleumer; 18, subsect. *Saluenensia* (Hutch.) Sleumer; 19, subsect. *Glauca* (Hutch.) Sleumer; 20, subsect. *Triflora* (Hutch.) Sleumer; 21, subsect. *Lanata* Chamb; 22, subsect. *Campylogyna* (Hutch.) Sleumer.

In addition, a series of ANOVAs were conducted to assess the significance of these factors. We used the Type III sum of squares to establish the significance level of each effect because of the unbalanced data. The main effect of each factor on the variance of PFG was estimated by one‐way ANOVAs. Next analysis of deviance was used to test the independent effect of each factor. Comparing the full model (e.g., species+seed mass+plant height+habitat+altitude) with different reduced models (seed mass+plant height+habitat+altitude), the difference between the proportion of the total sum of squares (SS) explained by the *R* ^2^ of the full model and the *R* ^2^ of the reduced model explained the independent effect by the deleted variable.

GLM was applied to achieve proportion of the variation, and each character can account for when every character was first explanatory.

Seed mass: Each species was assigned to a seed mass class: 0–0.05, 0.05–0.10, 0.15–0.20, and 0.20–0.25 mg.

Habitat: Each species was assigned to a habitat category based on the site of seed collection in either alpine shrub, rocky slope, or forest.

Altitude: Each species was assigned to an altitude class (2,500–3,000, 3,000–3,500, 3,500–4,000, and >4,000 m) developed based on temperature and vegetation distribution along the altitude gradient.

### 2.4 Statistical analysis

The average germination percentage of 36 *Rhododendron* species were calculated at each temperature under light and dark conditions. To explore the effect of altitude, habitat, seed mass, taxonomic level, and plant height on seed germination, data from the 10/20°C (light) were used to explore the effect of altitude, habitat, seed mass, taxonomic level, and plant height on seed germination. The reason 10/20°C regime was chosen was that for most species, the seeds germinated well in this temperature regime comparing with other temperature regimes according to our previous experiments.

Two indexes were used to evaluate seed germination: final germination percentage (FGP) and GR. The formula (Baskin, Baskin, & Chester, 2004) was as follows:

(1) $$\sum \frac{100G_{i}}{nt_{i}}$$

where *n* represented the number of seeds being processed and *G*_*i*_ represented the number of seeds germinating. A large GR value meant that seed germinated quickly (Lai et al., 2010; Zheng et al., 2008). All statistical analyses, including the test for homogeneity of variance, were performed using the SPSS 18.0 package (SPSS, Chicago, IL, USA).

## 3 RESULTS

### 3.1 Germination responses to temperature and light

**Figure 2:**
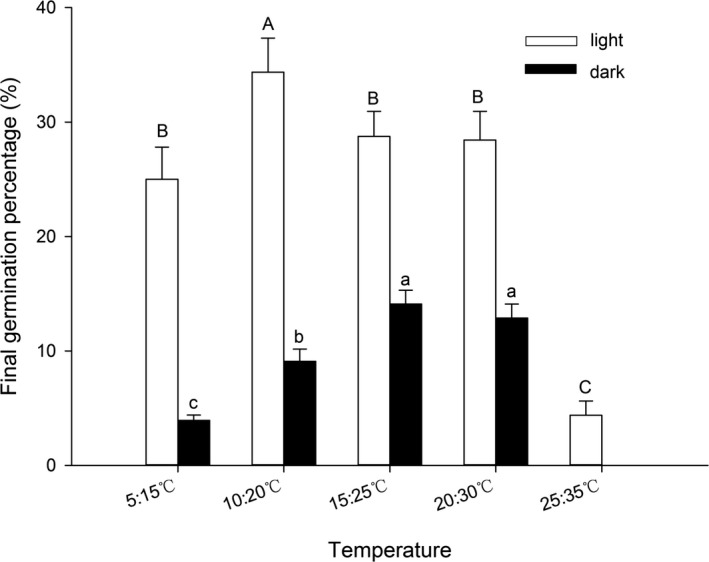
Final germination percentage (mean ± *SE*) of 36 Rhododendron species under different temperature and light conditions. Each bar represents all species within each group; bars with different letters are significantly different from each other at *p* < .05 (Turkey test)

Temperature, light, and the interaction between them had a significant effect on seed germination (Table 2). Average FGP and GR were higher in the light than in the dark (Figure 2). In the light, the average FGP was the highest at 10/20°C and the lowest at 25/35°C, and GR was the highest at 15/25°C and the lowest at 5/15°C. In the dark, the average FGP was the highest at 15/25°C, and seeds could not germinate at 25/35°C.

**Table 2:** Results of two‐way ANOVA on final germination percentage (FGP) of the 36 *Rhododendron* species seeds under different temperature and light intensity

Source	*df*	FPG	
		*F*‐value	*p*‐value
Temperature (T)	4	13.478	.000
Light (L)	1	68.526	.000
T × L	4	4.363	.004

### 3.2 Germination responses to altitude

**Figure 3:**
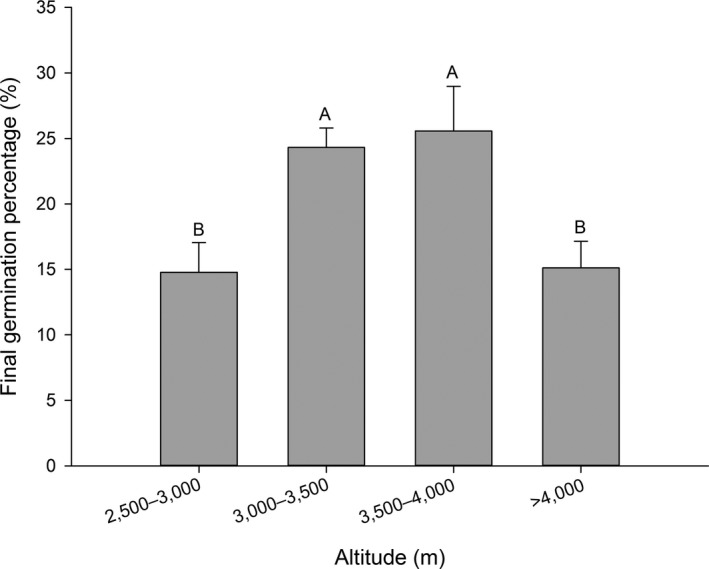
Final germination percentage (mean ± *SE*) of 36 Rhododendron species at different altitude. Each bar represents all species within each group.; bars with different letters are significantly different from each other at *p* < .05 (Turkey test)

Final germination percentage increased as altitude rose and reached the highest values of 26.7% and 33.5%, respectively, at 3,500–4,000 m then decreased as altitude increased further (Figure 3).

### 3.3 Germination responses to habitat

**Figure 4:**
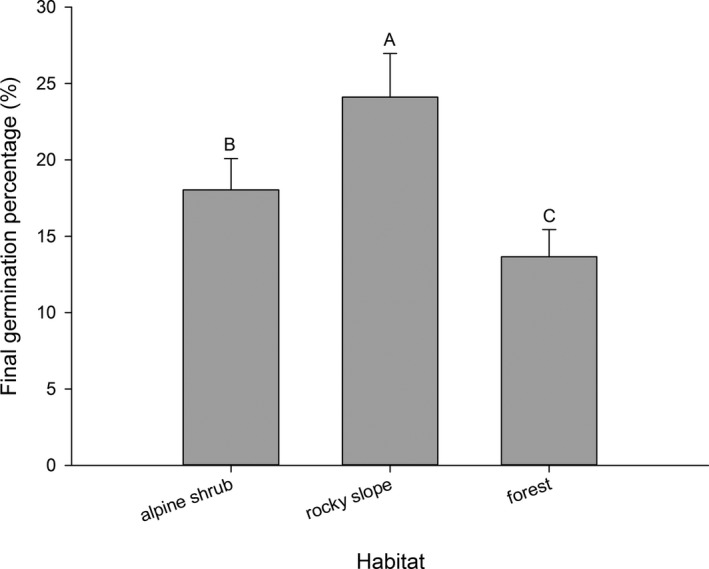
Final germination percentage (mean ± *SE*) of 36 Rhododendron species distributed in different habitats. Each bar represents all species within each group; bars with different letters are significantly different from each other at *p* < .05 (Turkey test)

Final germination percentage was the highest in the rocky slope habitat, the second highest in alpine shrub, and the lowest in forest (Figure 4).

### 3.4 Germination responses to plant height

**Figure 5:**
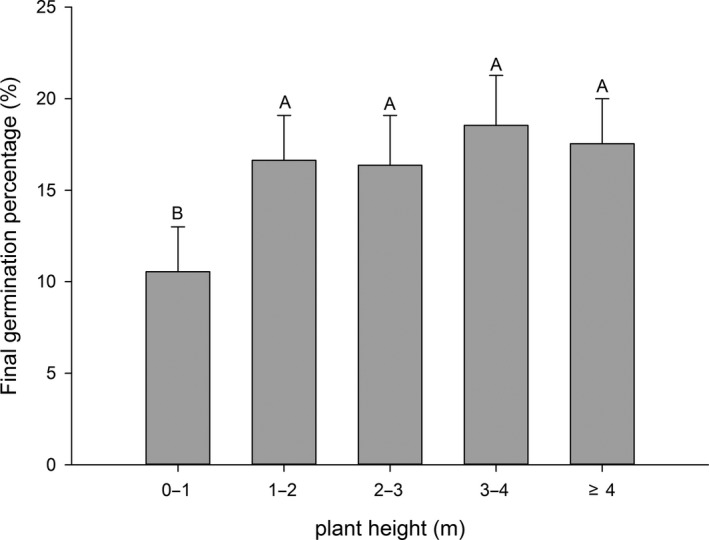
Relationships between seed mass and final seed germination percentage and germination rate of 36 Rhododendron plants. Each bar represents all species within each group; bars with different letters are significantly different from each other at *p* < .05 (Turkey test)

Final seed germination percentage and rate of plants above 1 m were significantly higher than those of <1 m (Figure 5).

### 3.5 Germination responses to seed mass

**Figure 6:**
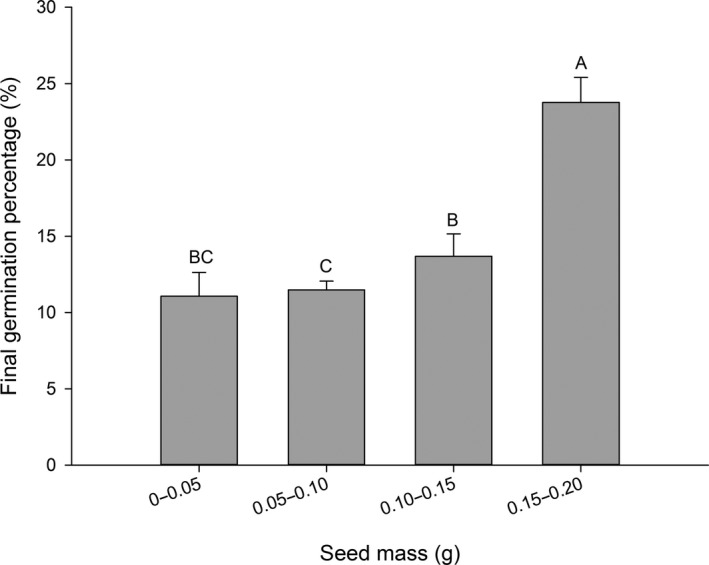
Final germination percentage (mean ± *SE*) of 36 Rhododendron species with different plant height. Each bar represents all species within each group; bars with different letters are significantly different from each other at *p* < .05 (Turkey test)

Final germination percentage rose as seed mass increased. At thousand‐seed masses of 0.15–0.20 g, FGP was high with values of 24.2% and 24.6%, respectively (Figure 6).

### 3.6 Germination responses to phylogeny

When all taxonomic levels from genus to species together were considered, taxonomic membership accounted for 69% of FPG variation (Table 3). The results showed that FPG is related to phylogeny. Most seed germination variation was associated with differences among species within subsection and differences among subsection within section. Variation among sections within subgenera and variation among subgenera within genus contributed little possibly because there were few subgenus per genus and sections per subgenera (Table 3).

**Table 3:** Total amount of FGP explained by a nested model including all taxonomic levels above species; component *R* ^2^, FGP variation explained by subgenus, section within subgenus, subsection within section, species within subsection

Source of variance	*df*	Sum of squares	Component *R* ^*2*^
Total	147	52.25	1
Subgenus	1	5.61	.1
Section	2	7.93	.14
Subsection	21	8.42	.17
Species	36	14.46	.28
Error	87	15.83	.31

According to the GLM, taxonomic species accounted for the large proportion of the variation in FPG when it was used as the first explanatory variable (38%) (Table 4). By contrast, when comparing the full model with different reduced models, we found that the percentage of variance in FPG explained by species membership independently decreased to 30% when removing the effects of other factors, but the percentage of variance in FPG explained by seed mass independently only decreased 2% when removing the effects of other factors. The other plant traits could only account for independently 4% (altitude), 7% (habitat), and 8% (plant height) (Table 5).

**Table 4:** Power of each factor to explain FGP variation in GLM containing a categorical variable

Source	*df*	*MS*	*F*	*R* ^*2*^
Species	35	0.43	9.45	.38
Seed mass	3	1.87	38.41	.12
Plant height	4	0.95	19.76	.06
Habitat	2	1.75	36.42	.09
Altitude	3	0.89	17.93	.07

**Table 5:** Multifactorial ANOVAs for the independent effects of each factor. To calculate the proportion of the variance explained by only one of the main factors, we subtracted the *R* ^2^ of the incomplete ANOVA with that factor removed, from the *R* ^2^ of the complete model

Source of variation		Full model				Plant height removed		
	*df*	*MS*	*F*	*R* ^2^	*df*	*MS*	*F*	*R* ^2^
Species	35	0.31	7.13	.35	35	0.34	7.12	.36
Seed mass	3	0.24	6.47	.15	3	0.26	6.51	.16
Plant height	4	0.14	3.42	.06				
Habitat	2	0.09	2.16	.03	2	0.10	2.26	.04
Altitude	3	0.12	2.54	.07	3	0.14	2.56	.08
Corrected model	47	0.38	9.16	.41	43	0.39	9.26	.33

		Species removed				Habitat removed		
		*MS*	*F*			*MS*	*F*	
Species					35	0.36	7.15	.37
Seed mass	3	1.10	6.47	.28	3	0.28	654	.17
Plant height	4	0.42	9.36	.10	4	0.18	3.44	.07
Habitat	2	0.21	5.38	.05				
Altitude	3	0.35	7.12	.12	3	0.15	2.61	.13
Corrected model	12	0.62	10.38	.11	45	0.39	9.21	.34

		Seed mass removed				Altitude removed		
		*MS*	*F*			*MS*	*F*	
Species	35	0.36	7.52	.37	35	0.33	7.21	.36
Seed mass					3	0.27	6.52	.17
Plant height	4	0.17	3.58	.09	4	0.16	3.48	.08
Habitat	2	0.13	2.31	.05	2	0.12	2.22	.05
Altitude	3	0.15	2.69	.12				
Corrected model	47	0.41	9.42	.31	44	0.43	10.23	.37

## 4 DISCUSSION

### 4.1 Relationship of seed germination with temperature, light, and altitude

In the life cycle of a plant, seeds have the highest resistance to extreme environmental stresses (Motsa, Slabbert, van Averbeke, & Morey, 2015). Successful establishment of a plant population is dependent on the adaptive aspects of seed germination (Qu, Huang, Baskin, & Baskin, 2008).

The FGP of *Rhododendron* species were found to be significantly affected by environmental factors. Temperature, light, and their interaction had obvious influences on seed germination, and FGP in the light was better than in the dark. The effects of dry storage at 5°C could have induced physiological changes to break innate dormancy (Baskin & Baskin, 2014b). Germination percentage and rate could be influenced by the altitude at which seed was collected. In this study, seeds of *Rhododendron* collected at the highest sites reached higher germination percentages and rates than those from the lower sites. Chilling followed by favorable temperatures represents a natural dormancy‐breaking mechanism in many species (Mamo, Mihretu, Fekadu, Tigabu, & Teketay, 2006). Seeds collected by us at the higher altitudes might have started the stratification period earlier than seeds from lower sites because of the reduced temperature as altitude increased. Seeds of some *Rhododendron* species were dormant before germination. Many factors can break down seed dormancy, of which low temperature is an important one (Eeckhaut, De Keyser, Van Huylenbroeck, De Riek, & Van Bockstaele, 2007). This may explain why *Rhododendron* seeds from high‐altitude populations, subjected to low temperatures, showed higher germination percentages. In southern France, Cummins and Miller (2003) reported similar results: seeds from high sites germinated better than those collected in low sites. This may partly be explained by an earlier and higher germination of seeds from high sites when subjected to optimal temperatures for germination during a brief growing season. An earlier germination would facilitate the emergence and survival of seedlings in the first phases of the life cycle in the mountain site (McGraw and Vavrek 1989).

### 4.2 Relationship between seed germination and seed mass

Seed masses of angiosperm species range over 11.5 orders of magnitude from the seeds of orchids, some of which weigh just 0.0001 mg like dust to the 20‐kg seeds like coconut. Seed mass has effects on many aspects of plant ecology. Seed mass was in connection with environmental factors. Along the gradient of altitude, seed mass varies significantly. Guo, Mazer, & Du (2010) suggested a negative correlation between seed mass and altitude among populations on nine species. A decline in seed mass with altitude may be because of environmentally induced plastic response to a decline in resource availability. Low temperature at high altitude may reduce photosynthetic rates, and short growing seasons may reduce the time of seed development, reducing mature seed mass. Seed mass is in correlation with the ecological history of plants (Mols et al., 2005). Seedlings from large seeds have greater competitiveness and higher survival ability in terms of competition, shading, drought, and nutrient limitation, whereas small‐seeded species can produce more seeds for a given quantity of energy than large‐seeded species (Kahmen & Poschlod, 2008; Mols et al., 2005). Previous studies found different results on the relationship between seed germination and seed size. Some studies have found large seeds have a better capability of germinating and the surviving of seedling than small ones (Bu et al., 2007; Chen et al., 2013; Kahmen & Poschlod, 2008). Rosbakh and Poschlod (2015) found that seed size had no effect on seedlings and that seed germination was mainly driven by exterior environmental factors and not by internal factors including seed size. Others found that seed germination had no relationship with seed mass (Liu, Yan, Li, Ma, & Ling, 2007), while some researchers found that seed size had a negative effect on seed germination (Jankowska‐Blaszczuk & Daws, 2007). In our research, the seed mass had a significant positive effect on seed germination: big seeds had higher germination percentages both in the light and in the dark. These results could be due to the larger storage reserves in these seeds (Norden et al., 2009; Zhao, Mo, Wang, Zhang, & Long, 2015).

### 4.3 Relationship between seed germination and phylogeny

The effect of phylogeny on plant history is known as phylogenetic constraint (Baskin & Baskin, 2014a). Some researchers have concluded that phylogenetic constraint can explain the variation in speed of species evolution (Wang et al., 2009). In our study, we studied 36 species comprising one genus, 2 subgenera, 3 sections, and 23 subsections. At species level, there were significant variations in seed germination percentage and rate, but not at subsection, section, or subgenus levels. This showed that in the origin and evolution of *Rhododendron*, seed germination as a life history trait was constrained by phylogeny, and attributes of the latter have been retained. Our results of the relationship between seed germination and phylogeny were in agreement with the results of seed germination in an alpine meadow (Bu et al., 2007).

Seed dispersal mode and seed size had an intimate relationship with phylogeny. Genetic materials also played an important role in evolution; random factors and environment were also important selection pressures inducing phylogeny change (Baskin & Baskin, 2014b). We could not infer a direct influence of phylogeny on seed germination, because some ecology and life history factors were not considered. However, our results suggest that phylogeny and life cycle characteristics should be considered when studying seed germination.

### 4.4 Relationship between seed germination and plant height

Taller plants have a better dispersal (Hintze et al., 2013), which can be an adaptation to avoid enemies and maternal competition. It also increases the probability that an individual will find a suitable habitat (Vandelook & Van Assche, 2008). Plant height significantly influenced seed germination in this study, and seeds from tall plants had a high FGP. Seed dispersal can serve to spread risks in a patchy environment, to avoid competition between parent and offspring and to allow seeds to germinate successfully and establish themselves in a suitable place (Fenner & Thompson, 2005). Offspring location is dependent on the number of seeds dispersed at any distance from the parent (Janzen, 1970). Wind‐dispersed seeds travel further from tall plants than from short plants, which leads to lower germination percentages for tall plants. Numerous seeds being located together can result in aggregated seedling recruitment, resulting in sibling competition. Therefore, seeds from tall plants have greater germination percentages than those from short plants. Seed germinability was in accordance with plant height in this study. *Rhododendron* seeds are dispersed by wind, and tall plants assist seed dispersal. The dispersal success of seeds from low plants was poor, limited to the habitat near the maternal plant. This can result in sibling rivalry and decrease adaptation ability. Seeds of some *Rhododendron* species with poor dispersal ability might avoid individual competition by low germination.

## 5 CONCLUSIONS

The FGP of *Rhododendron* species was significantly affected by environmental factors and was higher in the light than in the dark. Germination rose as altitude increased and then decreased when the highest levels of germination had been reached. Larger seeds had higher FGPs no matter whether they were in the dark or in the light. Seed germination was affected by phylogeny, and there were significant variations in germination at species level but not at other levels. Taller plants had higher germination than lower plants.

## CONFLICT OF INTEREST

None declared.

## AUTHOR CONTRIBUTIONS

Yongji Wang, Yuanrun Zheng: Conceived and designed the experiments. Yongji Wang: Performed the experiments. Liming Lai, Hui Du, Lianhe Jiang: Analyzed the data. Wang Fei, Zhang Chao, Ping Zhuang: Contributions reagents/materials/analysis tools. Yongji Wang, Yuanrun Zheng: Wrote the paper.
